# Migraine in pregnancy

**DOI:** 10.1186/1129-2377-16-S1-A24

**Published:** 2015-09-28

**Authors:** Simona Sacco, Patrizia Ripa

**Affiliations:** Institute of Neurology, Department of Applied Clinical Sciences and Biotechnology, University of L'Aquila, L'Aquila, Italy

Migraine is a predominantly female disorder. Evidence suggests that migraine activity is influenced by hormonal factors, and particularly by estrogen levels and fluctuations. During pregnancy, estrogen may reach one hundred times the normal level, whilst progesterone levels decrease, rising again towards the end of the pregnancy; however, hormonal fluctuations are not as pronounced as during the non-pregnant state[1]. Most women with migraine report an improvement of their attacks during pregnancy, from the first to the third trimestre, particularly women with a history of menstrual migraine and with migraine without aura[2]. This improvement may be due to the lack of hormonal fluctuations but also to the increased levels of natural pain-killing hormones (endorphins) induced by pregnancy[1, 3]. If no improvement is seen toward the end of the first trimestre, migraine is likely to continue throughout pregnancy and postpartum. Most women with migraine improving in pregnancy will experience attack recurrence shortly after delivery, likely in the first weeks[2]. This decline might be due to the precipitous drop in estradiol and endorphin levels occurring in the postpartum period[1, 3]. A small number of pregnant women experience a worsening of their migraine while a few others may even develop *de novo* migraine symptoms. In this context, migraine usually occurs during the first trimestre and is most often with aura[2]. Women continuing to experience migraine attacks throughout pregnancy may require treatment but we need to consider that not all medications used for migraine are safe in pregnancy. Paracetamol is the preferred drug for acute treatment throughout pregnancy. If paracetamol is not sufficiently effective, sporadic use of sumatriptan can be considered. NSAIDs such as ibuprofen can also be used under certain circumstances, though their intake in the first and third trimestres is associated with specific risks and contraindications. For prevention, non pharmacological approaches are always first-line treatment, and should also be used to complement any drug treatment. Some vitamins and dietary supplements have been proposed, such as, magnesium, riboflavin and coenzyme Q10. Preventive drug treatment should only be considered in the most severe cases and should include low doses of β-blockers and amitriptyline[4]. A personal history of migraine headaches can affect pregnancy outcomes. There is increasing evidence showing that migraine is a risk factor for several vascular complications during pregnancy, including gestational hypertension and preeclampsia, stroke, myocardial infarction, and venous thromboembolism; therefore, migraine should be considered a potential cardiovascular risk factor in obstetric care[5]. Further research is warranted to understand the mechanisms underlying the increased risk of vascular disease in pregnant migraineurs. Better understanding of those mechanisms could lead to potential treatment and earlier intervention, thereby reducing the health care costs of morbidity and mortality associated with adverse vascular events in this population.
